# Establishment of epidemiological cutoff values for *Fonsecaea pedrosoi*, the primary etiologic agent of chromoblastomycosis, and eight antifungal medications

**DOI:** 10.1128/jcm.01903-24

**Published:** 2025-04-04

**Authors:** Dallas J. Smith, Marcia S. C. Melhem, Jessy Dirven, Conceição Maria Pedrozo e Silva de Azevedo, Sirlei Garcia Marques, Bruna Jacomel Favoreto de Souza Lima, Vania Aparecida Vicente, Maria da Glória Teixeira Sousa, James Venturini, Nathan P. Wiederhold, Amir Seyedmousavi, Philippe J. Dufresne, Sybren de Hoog, Shawn R. Lockhart, Ferry Hagen, Daniel Wagner de C. L. Santos

**Affiliations:** 1 Mycotic Diseases Branch, Centers for Disease Control and Prevention 1242 , Atlanta, Georgia, USA; 2 School of Medicine, Federal University of Mato Grosso do Sul 54534 , Campo Grande, State of Mato Grosso do Sul, Brazil; 3 Tropical Medicine Institute, Medical School, University of São Paulo 28133 , São Paulo, Brazil; 4 Mycology Unit, Parasitology and Mycology Department, São Paulo State University 28108 , São Paulo, Brazil; 5 Westerdijk Fungal Biodiversity Institute, Utrecht, The Netherlands; 6 Post-graduation Program of Health Science, Federal University of Maranhao 37892 , São Luís, Maranhão, Brazil; 7 Laboratório Cedro, São Luís, Maranhão, Brazil; 8 Department of Infectious Diseases and Infection Control, Universidade Federal do Maranhão, EBSERH, São Luís, Maranhão, Brazil; 9 Post-graduation Program of Microbiology, Parasitology and Pathology, Federal University of Parana 28122 , Curitiba, Brazil; 10 Radboudumc/CWZ Center of Expertise for Mycology, Nijmegen, The Netherlands; 11 Post-graduation Program of Engineering Bioprocess and Biotechnology, Federal University of Parana 28122 , Curitiba, Brazil; 12 Faculdade de Medicina FMUSP, Institute of Tropical Medicine, Universidade de Sao Paulo 28133 , São Paulo, State of São Paulo, Brazil; 13 Fungus Testing Laboratory, Department of Pathology and Laboratory Medicine, University of Texas Health Science Center 14742 , San Antonio, Texas, USA; 14 Microbiology Service, National Institutes of Health Clinical Center 24481 , Bethesda, Maryland, USA; 15 Laboratoire de santé publique du Québec, Institut National de Santé Publique du Québec (INSPQ) 54470 , Sainte-Anne-de-Bellevue, Québec, Canada; 16 Department of Medical Microbiology, University Medical Center Utrecht, Utrecht, The Netherlands; 17 Institute for Biodiversity and Ecosystem Dynamics, University of Amsterdam 1234 , Amsterdam, The Netherlands; 18 Instituto D´Or de Pesquisa e Ensino, IDOR, São Paulo, Brazil; University of Utah, Salt Lake City, Utah, USA

**Keywords:** chromoblastomycosis, *Fonsecaea pedrosoi*, neglected tropical disease, fungi, implantation mycosis, epidemiological cutoff value

## Abstract

Chromoblastomycosis, a fungal neglected tropical disease, is acquired through traumatic inoculation and is clinically characterized by a chronic granulomatous infection of the skin and subcutaneous tissue. *Fonsecaea pedrosoi* is the most commonly reported etiologic agent globally. Itraconazole is considered first-line therapy, but successful treatment with terbinafine, voriconazole, and posaconazole has been reported. *F. pedrosoi* minimum inhibitory concentration (MIC) data are limited, and epidemiological cutoffs (ECVs) are lacking; such data are important to help monitor antifungal resistance trends and guide initial antifungal selection. Thus, we performed antifungal susceptibility testing (AFST) on *F. pedrosoi* isolates and determined the MIC distributions and ECVs. AFST on *Fonsecaea pedrosoi* isolates was conducted at six laboratories from October 2023 to June 2024. Species identification was previously confirmed by DNA sequence analysis. AFST was performed by CLSI M38 standard broth microdilution method for itraconazole, voriconazole, posaconazole, isavuconazole, ketoconazole, terbinafine, flucytosine, and amphotericin B. The ECVs were established using the iterative statistical method with ECOFFinder (version 2.1) following CLSI M57 guidelines. We analyzed MIC results from 148 *Fonsecaea pedrosoi* isolates. The calculated ECVs were itraconazole, 0.5 µg/mL; voriconazole, 0.5 µg/mL; posaconazole, 0.5 µg/mL; isavuconazole, 1 µg/mL; ketoconazole, bimodal, no ECV determined; terbinafine, 0.25 µg/mL; flucytosine, rejected; and amphotericin, 8 µg/mL. These *Fonsecaea pedrosoi* ECVs, obtained through a multicenter international effort, provide a baseline to better understand the *in vitro* antifungal susceptibility profile of this species and monitor resistance. Clinicians and researchers can use these values to detect non-wild-type isolates with reduced susceptibility, reevaluate therapeutic options, and investigate potential clinical resistance if treatment failure occurs.

**IMPORTANCE:**

Chromoblastomycosis is a neglected tropical disease caused by an environmental, dematiaceous fungus. This fungal disease is acquired after a break in the skin that allows the fungus to enter, leading to a chronic infection in the skin and subcutaneous tissue. It is difficult to treat and often requires years of antifungal treatment. *Fonsecaea pedrosoi* is the most reported causative agent globally. Limited antifungal susceptibility data exist for *F. pedrosoi* making interpreting minimum inhibitory concentration (MIC) results difficult. We performed antifungal susceptibility testing on 148 *F*. *pedrosoi* isolates to establish MIC distributions and epidemiologic cutoff values (ECVs) for eight antifungals, including those commonly used to treat chromoblastomycosis. The calculated ECVs for the commonly used antifungals itraconazole and terbinafine were 0.5 and 0.25 µg/mL, respectively. ECVs can be helpful in choosing potential treatment options for *F. pedrosoi* and monitoring antifungal resistance epidemiology.

## INTRODUCTION

Chromoblastomycosis is an implantation mycosis acquired through traumatic inoculation and clinically characterized by a chronic granulomatous infection of the skin and subcutaneous tissue (1). This disfiguring fungal infection was added to the World Health Organization’s list of neglected tropical diseases in 2017, highlighting the neglect by scientific and public health communities and its disproportionate impact on impoverished populations (2). Chromoblastomycosis is caused by dematiaceous fungi, with *Fonsecaea pedrosoi* being the most commonly reported etiologic agent globally (1, 3).

Global treatment guidelines are not available for chromoblastomycosis, but prolonged therapy and surgery are often required, lasting for months to years. Itraconazole is commonly considered first-line therapy with response rates ranging from 15% to 80% (1, 4–6). Terbinafine is also used frequently to treat chromoblastomycosis and may offer advantages such as better absorption and fewer drug-drug interactions (7). Other triazole agents such as voriconazole and posaconazole have been reported to have favorable clinical responses in refractory disease (8–10). Combination therapy with itraconazole and terbinafine or flucytosine has been used in clinical practice for severe or refractory disease (11, 12). Over 78 million people globally may not have access to itraconazole, and clinicians may resort to using ketoconazole, which has severe hepatic toxicity risks (13, 14).

In the setting of global emergence and increase of antifungal resistance, monitoring for resistance is critical to inform clinical and public health guidance (15). Antifungal minimum inhibitory concentration (MIC) data for *F. pedrosoi* are limited as chromoblastomycosis occurs in resource-limited settings where antifungal susceptibility testing (AFST) is difficult to perform. Previous reports found relatively low MICs to itraconazole and other azoles, but these were a limited number of isolates and tested in single laboratories (16–19). One report examined sequential isolates of *F. pedrosoi* and reported both high MIC values and clinical resistance to itraconazole, likely from treatment pressure (20).

Epidemiological cutoff values (ECVs) determine if a fungal isolate is non-wild type with regard to its *in vitro* response to an antifungal (21). ECVs can be a useful tool to monitor antifungal resistance, especially in patients with long-term antifungal use, which is required for chromoblastomycosis caused by *Fonsecaea pedrosoi* (22). They are not intended to provide a replacement for breakpoints but may assist clinicians in making more rational decisions, as non-wild-type strains may not respond to a specific antifungal therapy the same way as a wild-type strain of the same species. ECVs do not predict therapeutic response but may help determine whether an alternative therapy, if available, should be considered. As determined as a priority by the global chromoblastomycosis strategy (23), we performed AFST on *F. pedrosoi* isolates and established their MIC distributions, ECVs, percentage of non-wild type, modal MICs, MIC_90_s, and geometric mean for the eight antifungals: itraconazole, voriconazole, posaconazole, isavuconazole, ketoconazole, terbinafine, flucytosine, and amphotericin B.

## RESULTS

Antifungal susceptibility testing was performed by broth microdilution as outlined in the CLSI reference standard M38 Ed3 for filamentous fungi (24) for 148 *F*. *pedrosoi* isolates from 12 countries in six different labs (Table 1). ECVs, using the iterative statistical method with ECOFFinder (version 2.1) following CLSI M57 guidelines (21), for each antifungal were 0.5 µg/mL for itraconazole, voriconazole, and posaconazole; 1 µg/mL for isavuconazole; 0.25 µg/mL for terbinafine; and 8 µg/mL for amphotericin B (Table 2). Ketoconazole had a bimodal MIC distribution, and an ECV could not be determined. The ECV for flucytosine was also not set due to marked variability in the MIC results.

**TABLE 1:** Minimum inhibitory concentrations for *Fonsecaea pedrosoi* and eight antifungal medications

Antifungal (number of isolates)^a^	Number of isolates per minimum inhibitory concentrations (μg/mL)											
	0.016	0.03	0.06	0.12	0.25	0.5	1	2	4	8	16	≥32
Itraconazole (147)		23	43	**48**	22	10	1					
Voriconazole (144)	2	3	38	**54**	36	9	2					
Posaconazole (143)	5	27	29	**47**	32	3						
Isavuconazole (145)	1	9	20	**52**	40	16	7					
Ketoconazole (134)		3	9	**35**	17	3	3	5	31	25	3	
Terbinafine (142)	8	36	40	**47**	10		1					
Flucytosine (145)		1		1	2	8	5	18	25	**37**	26	22
Amphotericin B (144)		1		1	10	16	**50**	46	20			

^a^ Some laboratories were unable to perform antifungal susceptibility testing on all antifungals due to inaccessibility to an antifungal or difficulties with an *F. pedrosoi* isolate. Modal MICs are in bold.

**TABLE 2:** Epidemiological cutoff values and minimum inhibitory concentration characteristics for *Fonsecaea pedrosoi* and eight antifungal medications^*c*^

	ECV (μg/mL)	% NWT	Modal MIC (μg/mL)	MIC_90_ (μg/mL)	Geometric mean MIC (μg/mL)
Itraconazole	0.5	0.7	0.12	0.5	0.10
Voriconazole	0.5	1.4	0.12	0.25	0.13
Posaconazole	0.5	0	0.12	0.25	0.09
Isavuconazole	1	0	0.12	0.5	0.16
Ketoconazole	Bimodal	N/A^*a*^	Bimodal	8	0.81
Terbinafine	0.25	0.7	0.12	0.12	0.07
Flucytosine	Not set	N/A^*a*^	8	32	5.94
Amphotericin B	8^*b*^	0	1	4	1.22

^*a*^ Percentage of non-wild type was not applicable for ketoconazole or flucytosine because no epidemiological cutoff values were established.

^*b*^ The amphotericin B epidemiological cutoff value for *Fonsecaea pedrosoi* is very high. A minimum inhibitory concentration value lower than the ECV does not imply that the isolate is susceptible to amphotericin B.

^*c*^ NWT, non-wild type.

The percentage of non-wild-type isolates (greater than the established ECV) was 0.7% (1/147) for itraconazole, 1.4% (2/144) for voriconazole, 0.7% (1/142) for terbinafine, and 0% for posaconazole, isavuconazole, and amphotericin B (Table 2). Modal MICs were 0.12 µg/mL for all antifungals except 8 µg/mL for flucytosine and 1 µg/mL for amphotericin B.

## DISCUSSION

Because there is neither clear treatment guidance nor clinical trial data, treatment of chromoblastomycosis caused by *F. pedrosoi* is challenging. The susceptibility profile of this fungus is still not well characterized due to limited *in vitro* antifungal susceptibility data. Through a global international effort, we were able to collect MIC data for over 100 isolates and establish ECVs for *F. pedrosoi* for commonly used antifungals and those reserved for refractory disease. Isolates from diverse geographical areas on six continents were tested in six independent laboratories, strengthening these results and applicability. We found that MICs and the percentages of non-wild-type isolates were generally low, indicating that the occurrence of molecular mechanisms of resistance is likely low for clinical *F. pedrosoi* isolates. These ECVs, which provide some context for interpreting an MIC value, are an additional tool in selecting antifungal therapy while also considering factors such as pharmacokinetics and pharmacodynamics, site of infection, lesion size, and patient’s health status (e.g., CARD9 mutations) (4, 25).

Establishing MIC distributions for *F. pedrosoi* and other chromoblastomycosis-causing fungi is the first step in the determination of both ECVs and breakpoints. The distribution gives an idea of whether the modal and highest MICs are achievable during the course of treatment. For isolates with high MICs, such as those seen for flucytosine in our study, therapeutic dosing may be difficult to achieve without causing toxicity and severe adverse events. The side-effect profile for flucytosine includes dose-related drug toxicity, which can lead to bone marrow suppression, hepatoxicity, gastrointestinal intolerance, and renal impairment (26). Interestingly, flucytosine has sparingly been used successfully for refractory, severe chromoblastomycosis without any reports of side effects (12, 27). An isolate with an MIC below the ECV does not indicate that an infection can be treated with the antifungal, especially when the ECV is high, as we see for amphotericin B.

Few studies have performed AFST on *F. pedrosoi*, but the MIC data for itraconazole and terbinafine in our study seem to trend higher than what has been previously reported. A study of 21 isolates reported a modal MIC of 0.12 µg/mL for itraconazole, similar to that seen in our study, but the range of MIC values was 0.03–0.25 μg/mL; our study showed that the highest MIC for itraconazole was three dilutions higher (17). Another study of five *F. pedrosoi* isolates reported a range from 0.25 to 1 μg/mL for itraconazole and 0.06–0.12 for terbinafine; the highest MIC for terbinafine was four dilutions higher in our study (16). Both studies used CLSI M38 methodology for AFST. The differences in the range could reflect the number of isolates and participating laboratories included in our study, where the other studies were conducted in one laboratory. Alternatively, differences in itraconazole MICs seen between these studies could reflect differences in the patients’ prior antifungal exposure, although we were unable to collect patient antifungal use data.

The establishment of ECVs for *F. pedrosoi* is critical to monitor antifungal resistance trends. A non-wild-type isolate may trigger a change in the drug of choice if other options are available. A high percentage of non-wild-type isolates may suggest that fungi may not respond to the antifungal as expected and consideration should be given to different treatment options. The percentage of non-wild-type isolates for itraconazole and terbinafine was low (0.7%) in our study. A previous study followed patients with chromoblastomycosis over time and collected sequential isolates for performing AFST (20). During treatment with itraconazole, the MICs of four out of six patients rose substantially between the first and second isolates, resulting in clinical resistance and treatment failure in two patients. The ECVs established here can help monitor for a change to non-wild type isolates, which could provide clues of treatment pressure leading to treatment failure as in the sequential isolate study or serve as early warning indicators of isolates with genetic changes from environmental acquisition of the fungus. Whole-genome sequencing of isolates with higher MICs could potentially identify genes of genetic mutations leading to these higher MICs from both treatment and environmental pressure (28).

ECVs for ketoconazole and flucytosine could not be determined in our study. For ketoconazole, the MICs presented in a bimodal distribution. Ketoconazole has substantial safety issues (e.g., liver toxicity) and should be avoided for the treatment of skin infections like chromoblastomycosis (14). However, ketoconazole is often the only antifungal available in many regions of the world where chromoblastomycosis is more common (e.g., low- and middle-income countries and rural areas). The authors, although hesitant to include ketoconazole in this study, acknowledged this access issue but could not establish an ECV. Due to the pH-dependent *in vitro* activity of flucytosine observed against other fungi, such as *Aspergillus*, and the variability in MICs reported by the laboratories in this study (29), an ECV for flucytosine was not set (30, 31). Flucytosine should only be used in combination with other antifungals for the treatment of chromoblastomycosis, and AFST is unlikely to provide useful data concerning its efficacy.

The interpretation of these data and ECVs is subject to at least five limitations. First, isolates were tested *in vitro*; the muriform cells seen during infection were not used as the inoculum and may respond differently to exposure to antifungals than hyphae and conidia (1). Second, tissue concentrations of certain antifungals (e.g., itraconazole and terbinafine) may be higher than those in serum. Thus, higher concentrations at the site of these infections may be achievable for some drugs. Third, *in vitro* MICs may not correlate with treatment outcomes. Comprehensive epidemiological and clinical studies can explore whether treatment failure is associated with higher MICs and the mechanism of resistance. Fourth, longer incubation periods were needed (4–6 days) for *F. pedrosoi* in our study than in established CLSI protocols. Fifth, isolate data from an additional lab were excluded due to high interlab variation, and another lab repeated AFST for their isolates due to discordance in inoculum quantification methodology. We propose that CLSI revisit its protocols for dematiaceous fungi like *F. pedrosoi* based on these limitations and our experience. Suggested updates include longer incubation periods (4–6 days), using an absorbance value of 0.15–0.17, conducting an external quality assessment for AFST on a set of strains, and depositing reference strains of *F. pedrosoi* with low and high MIC values in the CDC Antimicrobial Resistance Isolate Bank and/or public culture collections (e.g., CBS, JCM, ATCC, and IHEM) to allow laboratories to assess if their MICs are within expected ranges for this specific species (32).

### Conclusion

Chromoblastomycosis-causing fungi are neglected globally but are a substantial public health problem. ECVs were established for *F. pedrosoi* for common antifungals used in treatment, including itraconazole (ECV: 0.5 µg/mL) and terbinafine (ECV: 0.25 µg/mL), and can be helpful tools in choosing potential treatment options for *F. pedrosoi*. Global partnerships are needed for further work on neglected fungi including for ECV and ultimately breakpoint development.

## MATERIALS AND METHODS

### Isolates

Species identification was previously confirmed by DNA analysis using ITS or ITS and D1D2 sequencing. Among the 148 *F*. *pedrosoi* isolates, 87 came from Brazil, 18 from the USA and Puerto Rico, 16 from Mexico, 9 from China, 4 from Canada, 4 from Venezuela, 3 from Cuba, 1 from Paraguay, 1 from Libya, 1 from France, 1 from Uruguay, and 1 from the Netherlands. Two isolate locations were unknown. The countries where the isolates were tested do not necessarily represent the countries where the infections were acquired.

Isolate data were submitted by six different labs on three continents: 43 isolates were submitted by Westerdijk Fungal Biodiversity Institute (WI-KNAW), Utrecht, the Netherlands; 67 isolates were submitted by Laboratório de Doenças Infecciosas e Parasitárias of the University of Mato Grosso do Sul, Mato Grosso do Sul, Brazil; 17 isolates were from Microbiological Collections of Paraná Network (CMRP-Taxonline), Federal University of Paraná, Curitiba, Brazil; 10 isolates were from the Department of Laboratory Medicine, National Institute of Health, Bethesda, MD, USA; 7 isolates were from the Fungus Testing Laboratory, University of Texas Health Science Center, San Antonio, TX, USA; and 4 isolates were from Laboratoire de Santé Publique du Québec, Québec, Canada.

### Antifungal susceptibility testing

One hundred forty-eight *F. pedrosoi* isolates were used in this study. AFST was performed by broth microdilution as outlined in the CLSI reference standard M38 Ed3 for filamentous fungi (24). Isolates used as reference strains or quality controls were *Aspergillus fumigatus* ATCC MYA 3626, *Aspergillus flavus* ATCC 204304*, Candida parapsilosis* ATCC 22019 (=CBS 604), *Pichia kudriavzevii* ATCC 6258 (=CBS 573), and *Hamigera insecticola* ATCC MYA-3630; quality controls were within M38M51S CLSI ranges. Itraconazole, voriconazole, posaconazole, isavuconazole, ketoconazole, terbinafine, flucytosine, and amphotericin B were tested. The MICs were determined visually after 48–144 hours (2–6 days, depending on the growth rate of the individual isolate) of incubation at 35°C. Inoculum was adjusted to an absorbance of 530 nm at 0.15–0.17 and verified by hemocytometer counting to be within the range of 0.2–2.5 × 10^6^ conidia/mL or by Cellometer X2 (Nexcelom, Manchester, UK) to perform cell counts and used that to prepare the inoculum that was within the range for further processing. The antifungal susceptibility endpoints for itraconazole, voriconazole, posaconazole, isavuconazole, ketoconazole, terbinafine, and amphotericin B were 100% inhibition of growth. For flucytosine, ≥50% inhibition of growth compared to the positive growth control was used.

### Epidemiological cutoff values

The ECV was established using the iterative statistical method with ECOFFinder (version 2.1) with a 97.5% threshold following CLSI M57 guidelines (21, 33). The percentage of non-wild type, modal MIC, MIC_90_, and geometric mean were calculated for each antifungal.

## AFTER EPUB

[This article was published on 4 April 2025 with incorrect spelling of the author, Amir Seyedmousavi. The spelling was corrected in the current version, posted on 21 April 2025.]
